# Synthetic benchmarks for machine olfaction: Classification, segmentation and sensor damage^☆^

**DOI:** 10.1016/j.dib.2015.02.011

**Published:** 2015-02-27

**Authors:** Andrey Ziyatdinov, Alexandre Perera

**Affiliations:** a B2SLab, Department of ESAII, Universitat Politenica de Catalunya, Pau Gargallo 5, Barcelona, Spain; b Centro de Investigacion Biomedica en Red en Bioingenierıa, Biomateriales y Nanomedicina (CIBER-BBN), Barcelona, Spain

## Abstract

The design of the signal and data processing algorithms requires a validation stage and some data relevant for a validation procedure. While the practice to share public data sets and make use of them is a recent and still on-going activity in the community, the synthetic benchmarks presented here are an option for the researches, who need data for testing and comparing the algorithms under development. The collection of synthetic benchmark data sets were generated for classification, segmentation and sensor damage scenarios, each defined at 5 difficulty levels. The published data are related to the data simulation tool, which was used to create a virtual array of 1020 sensors with a default set of parameters [1].

*The data presented here are publicly available at the web server of Polytechnic University of Catalonia on the following link* 〈*http://neurochem.sisbio.recerca.upc.edu/public/datasets/benchmarks*〉.

**Specifications Table**

**Table:** 

Subject area	*Chemistry, Engineering*
More specific subject area	*Chemometrics, Machine Olfaction, Electronic Nose, Chemical Sensing, Machine Learning*
Type of data	*Table*
How data was acquired	*Data simulation tool*
Data format	*Raw*
Experimental factors	*Statistical models used in the data simulation tool were fitted to a reference data set.*
Experimental features	*An array of 1020 virtual sensors was created by the data simulation tool with the default parameters. The sensor signals were generated in response to a rectangular gas pulse of 60 time units.*
Data source location	*Barcelona, Spain*
Data accessibility	*The data sets are publicly available at the web server of Polytechnic University of Catalonia on the following link**http://neurochem.sisbio.recerca.upc.edu/public/datasets/benchmarks*.

**Value of the data**

• The benchmark material in the field of machine olfaction was published for the first time.
• The proposed definitions of scenarios combined with the data simulation tool can be used as a reference workflow for other scenarios in machine olfaction.
• The generated data sets have concentration profiles of mixtures of analytes, a considerably large number of sensors and realistic noise in the data.

## 1 Materials and methods

Synthetic benchmarks were an alternative to the real measurements at the middle stage of the Neurochem project, when the main sensor array of the project was under development [2]. The realization of the synthetic experiments required a model of an array of gas sensors. That model needed to capture the main features shown by polymer sensors (the reference data set was measured with an array of conducting polymer sensors) and be simple enough so that it could be included in the system software. The model was implemented in the data simulation tool (the R package chemosensors) [1,3].

The synthetic benchmarks produced for the three scenarios classification, segmentation and sensor damage possess a particular feature of the large number of sensors (1020). This feature will particularly suit for examination of the role of diversity and redundancy among the sensors at large scale. Recent examples of the data analysis based on real large sensor arrays include an array of 96 metal-oxide sensors combined with 10 different sensor families modulated in temperature [5], and an array of 16,384 conducting polymer sensors based on 24 different kinds of polymer materials [6] (both arrays are products of the Neurochem project).

### 1.1 Scenarios

Ten scenarios for machine olfaction – classification, quantification, segmentation, habituation, event detection, novelty detection, drift compensation I, drift compensation II, sensor replacement I and sensor replacement II – were designed and formalized in the framework of the data simulation tool [3, Supporting Information, File S1]. For three of these scenarios – classification, segmentation, and sensor damage (adopted from sensor replacement scenario) – synthetic benchmark data sets at different difficulty levels were generated.

General definitions of the three scenarios are the following.

1. Classification scenario: John has three vessels with three odors A, B, C. The system is trained with all three compounds separately. John approaches the vessel B to the system. The machine identifies correctly odor B. The difficulty is the similarity between the odors to be identified.
2. Segmentation scenario: John has three vessels with three odors A, B and C. The system is trained with all three compounds separately. John approaches vessel B to the system. The machine identifies correctly odor B. John approaches A+B to the system. The machine identifies A and B sequentially. The difficulty is the similarity between the odors to be segmented.
3. Sensor damage: John has three vessels with three odors A, B and C. The system is trained with all three compounds separately. John approaches vessel B to the system. The machine identifies correctly odor B. A certain proportion of specific sensors in the array are (virtually) damaged. John approaches vessel B to the system. The machine identifies correctly odor B without new training. The difficulty is the proportion of sensors to be replaced.

Binary mixtures of two analytes A and C from the data simulation tool are used as gas classes for the benchmarks [1]. One should not confuse these two analytes A and C with the odors mentioned in the scenario definitions above and named with the same letters A, B and C. The concentration of analytes in mixtures is given in dimensionless units from 0% to 100%. The 100% concentration corresponds to the maximum concentration of the analyte in the reference data set, and the simulated sensors are modeled to be in the saturation regime at a level higher than 100%.

The scenarios are parametrized by difficulty levels from 1 to 5. Each scenario is described in terms of composition of gas classes in training and validation sets, and scenario difficulty. Table 1 reports these parameters of the scenarios.

For classification scenario, the difficulty is defined as the similarity between gas classes, which is the similarity between two analytes A and C in mixtures. Such definition of the scenario difficulty is independent of simulation models for data generation.

For segmentation scenario, the difficulty is determined as the similarity between the odors to be segmented. The closer the odors, the more difficult will be the task of mixture segmentation. One should note that the synthetic sensors have more affinity to analyte A in respect to analyte C, as the same relationship was observed in the reference data set. Hence, the increment in the difficulty level corresponds to a larger portion of analyte A in mixture in validation set.

For sensor damage scenario, the difficulty is defined by the proportion of damaged sensors in the array that were simulated to not respond in validation set. The signals from damaged sensors will be set to a baseline level with a small portion of the Gaussian noise (the noise is needed for data visualization in the multivariate space). The data sets from classification scenario at difficulty 3 were reused.

### 1.2 Data simulation tool

The reference dataset was obtained the facilities of the University of Manchester. Three gases at different concentration level were measured: ammonia (1%, 2%, 5%), propanoic acid (1%, 2%, 5%), n-butanol (1%, 10%). The experiments were repeated on a regular basis during 10 months. The sensor array was composed by 17 polymeric sensors. A total number of 3925 were acquired and labeled to mentioned gases and concentrations. The response of the sensors has 329 s time-length, sampled at 1 Hz frequency. The compound is induced to the sensor array at instant *t*=0 s, then the clean air enters the chamber at instant *t*=185 s. The detailed information about the UNIMAN data set and list of related applications can be found in [4] and references therein.

The simulation models were designed for polymer based gas sensors and validated on the reference data set of seventeen sensors described above [1]. The data simulation flow took a matrix of concentrations as input and returned a matrix of sensor array data as output. Two sorption and calibration models emulate the sensor response under noise-free conditions. Three models, concentration noise, sensor noise and drift noise, generate the noise in data at different stages of the simulation flow. The response of a single sensor to a mixture of analytes is controlled by the Langmuir isotherm, implemented in the sorption model. The Langmuir isotherm implies a competitive sorption behavior and results in a non-linear response. The complete description of the models is available in [1], and examples of data simulations by means of the data simulation tool are presented in [3].

Only one array of 1020 virtual sensors was created by the data simulation tool under version 0.4.3, in order to produce all the benchmark data sets for three scenarios. Since the reference data set was measured based on the array of seventeen polymeric sensors, each virtual sensor or sensor model was derived from a particular sensor prototype or reference sensor (the number of sensor types is seventeen). For the produced 1020 sensors, the sensor type is determined by the arithmetic operation of integer division, where numerator is the sensor index (the column index in the data tables) and denominator is seventeen. For instance, the sensors derived from the first reference sensor have indexes 1, 18, 35 and so on. One may consider working with a subset of the 1020 virtual sensors by selecting certain columns of the data tables and appropriately controlling the sensor types.

## 2 Data format

The benchmark data are distributed in comma-separated value format (csv). Some basic description for each data set is also distributed in automated report files given in PDF format. The delimiter between fields in the csv files is the “,” symbol. The approximate size of the data set for a single scenario is 100 Mb (50 Mb in zip compression).

The data tables have the following columns:

• ‘s1’, ‘s2’,… ‘s1020’: the transient signal values from 1020 sensors;
• ‘Gas’: the gas class label;
• ‘Set’: the set label, training, validation or interim (inter-medium set of samples between training and validation sets);
• ‘cA’, ‘cB’ and ‘cC’: the concentration values of analytes A, B and C, respectively;
• ‘time’: the time in abstract time units.

## Figures and Tables

**Table 1:** Description of benchmark data sets for three scenarios: classification, segmentation and sensor damage. Scenario difficulty, gas classes in training and validation sets are reported for each scenario. The number of samples per class in both training and validation sets is 30. For sensor damage scenario, the difficulty is defined by the proportion of damaged sensors in the array that were simulated to not respond in the validation set.

Classification			Segmentation			Sensor Damage		
Difficulty	Classes (T)	Classes (V)	Difficulty	Classes (T)	Classes (V)	Difficulty	Classes (T)	Classes (V)
1	A and C	A and C	1	A and C	A50C50	1 (6.25%)	A33C67 and A67C33	A33C67 and A67C33
2	A17C83 and A83C17	A17C83 and A83C17	2	A and C	A45C55	2 (12.5%)	A33C67 and A67C33	A33C67 and A67C33
3	A33C67 and A67C33	A33C67 and A67C33	3	A and C	A60C40	3 (18.75%)	A33C67 and A67C33	A33C67 and A67C33
4	A40C60 and A60C40	A40C60 and A60C40	4	A and C	A67C33	4 (25%)	A33C67 and A67C33	A33C67 and A67C33
5	A45C55 and A55C45	A45C55 and A55C45	5	A and C	A83C17	5 (31.25%)	A33C67 and A67C33	A33C67 and A67C33
